# Using population-based data to evaluate the impact of adherence to endocrine therapy on survival in breast cancer through the web-application BreCanSurvPred

**DOI:** 10.1038/s41598-022-12228-y

**Published:** 2022-05-16

**Authors:** Rebeca Font, Maria Buxó, Alberto Ameijide, José Miguel Martínez, Rafael Marcos-Gragera, Marià Carulla, Montse Puigdemont, Mireia Vilardell, Sergi Civit, Gema Viñas, Josep A. Espinàs, Jaume Galceran, Ángel Izquierdo, Josep M. Borràs, Ramon Clèries

**Affiliations:** 1 Pla Director d’Oncología, IDIBELL, Av. Gran Vía 199-203, 08908 Hospitalet de Llobregat, Barcelona, Spain; 2 Institut d’Investigació Biomèdica de Bellvitge, IDIBELL, Av. Gran Via de L’Hospitalet, 199-203 – 1a planta, 08908 Hospitalet de Llobregat, Barcelona, Spain; 3 Institut d’Investigació Biomèdica de Girona, IDIBGI, C/Dr.Castany S/N. Edifici M2. Parc Hospitalari Martí I Julià, 17190 Salt, Spain; 4 Registre de Càncer de Tarragona, Servei d’Epidemiologia i Prevenció del Càncer, Hospital Universitari Sant Joan de Reus, IISPV, Reus, Spain; 5 Department de Estadística I Investigació Operativa de La Universitat Politècnica de Catalunya. EDIFICI H, Diagonal 647, 08028 Barcelona, Spain; 6 Grupo de Investigación en Salud Pública, Universidad de Alicante, 03690 Alicante, Spain; 7 Registre de Cáncer de Girona – Unitat d’Epidemiologia. Pla Director d’Oncologia. Institut Català d’Oncología. Grup d’Epidemiologia Descriptiva, Genètica I Prevenció del Càncer de Girona-IDIBGI, 17005 Girona, Spain; 8 Facultat de Medicina, Universitat de Girona (UdG), Girona, Spain; 9 Centro de Investigación Biomédica en Red: Epidemiología y Salud Pública (CIBERESP), Madrid, Spain; 10 Independent Researcher, Barcelona, Spain; 11 Secció de Estadística del Departament de Genètica, Microbiología i Estadística de La Facultat de Biologia. Universitat de Barcelona, 08028 Barcelona, Spain; 12 Servei d’Oncología Médica, Institut Català d’Oncología. Hospital Universitari de Girona Doctor Josep Trueta, 17005 Girona, Spain; 13 Department de Ciències Clíniques de La Universitat de Barcelona, 08907 Barcelona, Spain

**Keywords:** Cancer, Breast cancer, Cancer epidemiology

## Abstract

We show how the use and interpretation of population-based cancer survival indicators can help oncologists talk with breast cancer (BC) patients about the relationship between their prognosis and their adherence to endocrine therapy (ET). The study population comprised a population-based cohort of estrogen receptor positive BC patients (*N* = 1268) diagnosed in Girona and Tarragona (Northeastern Spain) and classified according to HER2 status (+ / −), stage at diagnosis (I/II/III) and five-year cumulative adherence rate (adherent > 80%; non-adherent ≤ 80%). Cox regression analysis was performed to identify significant prognostic factors for overall survival, whereas relative survival (RS) was used to estimate the crude probability of death due to BC (P_BC_). Stage and adherence to ET were the significant factors for predicting all-cause mortality. Compared to stage I, risk of death increased in stage II (hazard ratio [HR] 2.24, 95% confidence interval [CI]: 1.51–3.30) and stage III (HR 5.11, 95% CI 3.46–7.51), and it decreased with adherence to ET (HR 0.57, 95% CI 0.41–0.59). P_BC_ differences were higher in non-adherent patients compared to adherent ones and increased across stages: stage I: 6.61% (95% CI 0.05–13.20); stage II: 9.77% (95% CI 0.59–19.01), and stage III: 22.31% (95% CI 6.34–38.45). The age-adjusted survival curves derived from this modeling were implemented in the web application BreCanSurvPred (https://pdocomputation.snpstats.net/BreCanSurvPred). Web applications like BreCanSurvPred can help oncologists discuss the consequences of non-adherence to prescribed ET with patients.

## Introduction

Breast cancer (BC) is the most common cancer in women worldwide in terms of incidence and mortality^1^. Traditionally, stage at diagnosis has been considered the most important factor for predicting survival in people with BC^2^. Other predictors, such as immunohistochemistry (IHC) tumor markers^3^, are also used for guiding treatment decisions^4^. In fact, IHC classification into BC subtypes based on hormone receptors (HR) such as estrogen and progesterone receptors, human epidermal growth factor receptor (HER2) and Ki67 is crucial for predicting BC survival^5,6^.

BC has four major subtypes according to the presence or absence of the three standard molecular markers (estrogen or progesterone receptors and HER2): HR + /HER2 − , HR + /HER2 + , HER2-enriched (HR − and HER2 +), and triple-negative (absence of the three standard molecular markers)^7^. Precision medicine for BC is a specific research area, where the genetic profile (HR + , HER2 + , and triple negative) informs the choice of the specific chemotherapeutic agent and individual treatments^7,8^. Systemic therapy relies on targeted drugs for achieving adequate effects: (i) endocrine therapies employing tamoxifen and/or aromatase inhibitors are the schedule for targeted treatment of HR + BC; (ii) a minority also receive chemotherapy; (iii) HER2 + tumors are treated with chemotherapy with HER2-targeted antibodies or small-molecule inhibitor therapy, and (iv) triple-negative tumors receive chemotherapy alone^7,8^.

For estrogen receptor positive BCs in early stage, five years of adjuvant endocrine treatment (ET) has been indicated as the reference treatment^7–14^. Tamoxifen for premenopausal women and aromatase inhibitors for postmenopausal women have shown their impact in reducing the risk of recurrence (almost 50%) and mortality (between 30 and 40%) during the first 10 to 15 years after initiation of treatment^7–9,13,14^. Despite these benefits, these tumors still carry a significant risk of late recurrence and death^7,8^. Clinical trials have shown a benefit of extending the ET up to 10 years, since the risks of cancer recurrence and in absolute BC mortality in women who extended ET can be lower than those of women who stopped ET at 5 years^7–9,13,14^. Extending tamoxifen treatment to 10 years showed a 4% higher reduction in the risk of recurrence and 2.8% reduction in BC mortality compared to the reductions in these risks found in women who stopped ET at 5 years^7,8^. In the case of aromatase inhibitors, usually prescribed to postmenopausal women, extending the treatment to 10 years has a higher reduction (between 3 and 4%) in the risk of disease recurrence compared to the reduction detected for those women who stopped the ET at 5 years^7,8^. There has also been some progress in combination therapies, which could inhibit tumor recurrence and might improve survival in BC patients^8^.

In population-based BC survival studies, age and stage at diagnosis are typically considered the basis for modeling BC survival^15–20^. However, treatment adherence, that is, “the extent to which a patient acts in accordance with the prescribed interval and dose of a dosing regimen”^21^, is not considered when assessing the population’s survival reference indicators, such as the crude probabilities of death^22–25^. Furthermore, the recent inclusion of the molecular subtype in some prediction models has improved predictive performance^16–20^. As noted above, tamoxifen and aromatase inhibitors are the pillars of adjuvant therapy for patients with HR + BC ^7,8^. Using population-based data, our team and others have also shown that non-adherence to ET is significantly and independently associated with recurrence and all-cause mortality after adjusting for age and tumor stage^18–20,26–34^. Thus, discontinuation of ET is related to negative impact in patient’s survival such as increased risk of (i) disease recurrence^18–20,26,27,31,34^, (ii) distant metastasis^26,29,30^, and/or (iii) mortality^18,26,28,30–34^.

In light of evidence suggesting that ET has survival benefits, it is recommended for at least five years^7,8,14,19,20^ to reduce the risk of recurrence and subsequent mortality^7^. Therefore, a manageable prognostic model for HR + BC that includes adherence to ET, age and stage could be of great interest for population-based BC survival statistics^19,20,22,23^, providing estimates of cause-specific survival indicators: the crude probabilities of death due to BC (P_BC_) and other causes (P_OC_)^24,25^. These population-based indicators provide a reliable estimate of cause-specific mortality, especially when these data are not directly available, a common situation in population-based cancer studies^25^. Our aim is to present these population-based statistical indicators that may be useful for clinical oncologists.

For the patient, a BC diagnosis sparks some important questions: “How much time is left for me? How do I manage with breast cancer? What is my annual/long-term prognosis?”. This paper shows how these indicators can be used in a web-based application to help clinicians make treatment decisions and talk to patients with HR + BC about the long-term impact of their active and voluntary adherence to ET for the first five years after BC diagnosis.

## Materials and methods

### The cohort dataset and the procedure for selecting the study population

BC data were obtained from population-based cancer registries in Girona and Tarragona (Catalonia, Spain), which covered an annual population of 771,854 women in 2007–2009. Follow-up was to 31 December 2019. In addition to the active and passive follow-up via hospitals, two passive follow-ups were undertaken using record linkage: one linking BC data with the Catalan Mortality Registry (which covers the four Catalan provinces of Girona, Tarragona, Lleida and Barcelona) and another linking data with the National Death Index of the Spanish Ministry of Health. Both mortality registries provided the date of death for all patients who died in Spain, but the specific cause of death could not be retrieved due to confidentiality protections. The patients not found to be dead at the end of follow-up were censored. Cancer registry data included 2049 women aged 20–84 years and diagnosed with invasive primary BC (International Classification of Diseases, 10th edition, code C50) from 2007 to 2009.

Medical records were reviewed to extract data on TNM stage at diagnosis for which we used the American Joint Committee on Cancer Staging manual 7th edition^35^. HR and HER2 overexpression were recorded from pathology and clinical reports. Table S1.1 of the supplementary material presents a simple descriptive of the whole cohort from Girona and Tarragona.

The first step in the study population’s selection, there were selected *N* = 1573 (HER2 − : *N* = 1185; HER2 + : *N* = 388) BC patients diagnosed with HR + BC in Girona and Tarragona database. Since ET is prescribed in patients diagnosed in stages I-III, there were excluded those patients diagnosed in stage IV or with missing stage at diagnosis. Therefore, a total of 1418 patients met that inclusion criterion. In these patients, adherence to ET was estimated based on the frequency of drug use during therapy, which in turn is related to the overall duration of the prescribed therapy (persistence)^21^. Adherence was assessed at five years from the date of the first prescription refill; prescriptions not refilled for more than two months were considered a discontinuation of therapy. Any switch to tamoxifen or aromatase inhibitor was considered a continuation of treatment. We estimated adherence as the proportion of days covered by a filled drug prescription over the treatment period (up to five years from the date of first prescription), deeming a cumulative adherence rate of 80% or more as satisfactory^18,20^. Data on ET prescription refills for BC were collected from 2007 to 2015 (covering at least five years for each BC patient) from the community pharmacy database, which is mandatory for drug reimbursement in Catalonia^19,20^. All included patients had received treatment at a public hospital, so the database included treatments for all patients. The date of recurrence or death, if occurring within five years of patient follow-up, was taken as the last date for calculating adherence. Using the cutoffs for the adherence rates validated in two previous studies^18,19^, patients were classified as adherent (adherence rate > 80%) or non-adherent (adherence rate ≤ 80%).

The second step was preparing the data for the analysis. There were excluded those patients with missing information on adherence to ET (*N* = 150, 32 out of 150 were estrogen receptor negative), leaving a total sample for the study population of *N* = 1268 BC patients with complete data. S1.2 of the supplementary material presents the patients characteristics of the data subsets used in these two steps of the procedure for selecting the study population.

### Study population (*N* = 1268)

Therefore, the study population used for the statistical analysis included all patients diagnosed before 85 years of age with estrogen receptor-positive BC in stages I, II or III and with complete information on adherence to ET during the years comprised between 2007 and 2019.

### Statistical analysis and development of the BreCanSurvPred web application

The variables considered for inclusion in the web application were age (assessed as both a continuous and a categorical variable, with age groups of ≤ 49 years, 50–59 years, 60–74 years, and 75–84 years), stage at diagnosis (I, II, III), adherence to ET (Yes: > 80%; NO: ≤ 80%), and molecular subtype (HER2 − , HER2 +).

Making use of competing risks modeling, which combines proportional hazards models for all-cause mortality and relative survival^24,25^, the P_BC_(T) and P_OC_(T) were calculated. Their analysis depends on the estimation of the λo(T), the overall hazard of death in the cohort at a specific time T, which can be obtained by means of a Cox model fitted to the cohort data^24^. The observed survival at any time T can be predicted as OS(T)=${\int }_{0}^{T}\mathrm{exp}[-{\lambda }_{O}\left(u\right)]du$ ^22–25^. Under additive modeling, the excess hazard of death in the cohort due to BC is defined as λ_X_(T) = λ_O_(T)-λ_P_(T), where λ_P_(T) is the expected hazard of death in the cohort according to the general population’s mortality rates^24^. From this quantity, the expected survival at time T is calculated ES(T)=${\int }_{0}^{T}\mathrm{exp}[-{\lambda }_{P}\left(u\right)]du .$ Finally, from the OS(T) and ES(T), we can estimate the RS(T) = OS(T)/ES(T), and from this quantity^24^, the five-year conditional relative survival (RS5), RS5(T) = RS(T + 5)/RS(T)^25^.

OS(T), P_BC_(T), P_OC_(T) and RS(T) are survival indicators which can be calculated up to 10 years according to the follow-up of our cohort. The indicator RS5(T) is another survival indicator of interest from a population-based perspective. It represents the patient’s five-year survival conditional on having survived, at least, T years after BC diagnosis and compared to the expected survival of the general population-cohort of the same age during the period of diagnosis^25^. Moreover, [1 − RS5(T)] × 100 can be used to estimate the “excess mortality”, EM of the patients compared to the risk in the general population ^36,37^. For instance, for a patient who has survived 3 years after BC diagnosis, RS5(3) is her additional five-year survival prediction compared to that of the general population. An RS5(3) of 0.95 would mean that the patient will have a 5% excess risk of death due to BC five years after the third year of follow-up. These RS5 estimates can help clinicians make decisions, for instance with regard to adjusting or personalizing treatments and determining patients’ long-term prognosis^25,37,38^. Note that, if RS5(T) was 1 for a certain T, that indicates no excess risk of death due to cancer.

In short, our modeling first estimates OS(T) and ES(T), and then uses these indicators to calculate P_BC_(T), P_OC_(T), RS(T), and RS5(T). The first step was to fit a Cox model to the study population’s dataset (patients with complete information for all variables) using all-cause mortality as the outcome. Four Cox models were assessed, with different assumptions according to age and adherence to ET: (i) model C.1 (age as categorical); (ii) model C.2 (age as log-linear continuous variable); (iii) model C.3 (age as continuous using restricted cubic splines^38,39^); and (iv) model C.4 (model C.3 but also considering adherence as a time-varying variable). The supplementary material presents an extension of the mathematical details and statistical methods used in this paper.

### Implementation of the survival indicators in BreCanSurvPred

The web application presents the estimated survival indicators for a BC patient according to age, stage, adherence to ET and molecular subtype at diagnosis. These are based on their predicted OS and the ES curves. The latter is calculated by applying the total hazard in the general population to each patient of the cohort, up to the maximum follow-up^38,40^, here 10 years after BC diagnosis. We used the age-specific all-cause mortality rates for 2007–2019 in Catalonia, the administrative region of Spain encompassing both cancer registries. Once the OS and the ES were calculated, the RS, RS5, P_BC_(T), and P_OC_(T) were derived. These indicators were all implemented in the web application BreCanSurvPred, available at https://pdocomputation.snpstats.net/BreCanSurvPred.

### Presentation of results

Chi-square tests were used to compare categorical variables, and t-tests to compare continuous variables (α = 0.05 in both cases) in tables. The plots showing differences in all-cause survival curves were adjusted for age through a Cox model^39,40^ and stratified according to the categories of each variable considered.

### Ethics declarations

All experimental protocols were approved by Comité de Ética de Investigación Clínica del Hospital Universitari de Bellvitge (Spain), Ethical approval number PR 160/18. All methods were carried out in accordance with relevant guidelines and regulations. Informed consent was obtained from all participants.

## Results

### Patient characteristics

Table 1 presents the baseline characteristics of 1268 patients with estrogen receptor + BC, according to molecular subtype (HER2 − : 75.3%; HER2 + : 24.7%). The cohort’s mean age was 58.4 years; 27.8% were under 50 years old, 59.6% were aged 50 to 74 years, 12.6% were 75 or older. Stage I was the most frequent among HER2 − patients (44.9%), whereas stage II was the most frequent among HER2 + patients (45.5%). A total of 1069 patients presented an adherence rate > 80%. Over 10-year follow-up, 18.1% of the patients died during, with almost half (10.7%) dying within the first five years. The mean length of follow-up was 9.3 years.

**Table 1:** Characteristics of patients diagnosed with hormone receptor-positive breast cancer before the age of 85 years in Girona and Tarragona, 2007 to 2009.

	HER2 − (*N* = 1185;75.3%)	HER2 + (*N* = 388;24.7%)	Total (*N* = 1573; 100%)	*p*-value
**Registry, *n* (%)**				
Girona	599 (50.5%)	208 (53.6%)	807 (51.3%)	0.29^a^
Tarragona	586 (49.5%)	180 (46.4%)	766 (48.7%)	
**Age**				
Mean (SD)	58.9 (12.9)	57.4 (13.2)	58.3 (13.1%)	0.87 ^b^
**Age groups, *n* (%)**				
0–49 years	329 (27.8%)	121 (31.2%)	450 (28.6%)	0.32^a^
50–59 years	291 (24.6%)	103 (26.5%)	394 (25.1%)	
60–74 years	389 (32.8%)	113 (29.1%)	502 (31.9%)	
75–84 years	176 (14.9%)	51 (13.1%)	227 (14.4%)	
**Stage**				
I	473 (39.9%)	116 (29.9%)	589 (37.4%)	< 0.05^a^
II	416 (35.1%)	160 (41.2%)	576 (36.6%)	
III	183 (15.4%)	70 (18.0%)	253 (16.1%)	
IV	57 (4.8%)	26 (6.7%)	83 (5.3%)	
Missing	56 (4.7%)	16 (4.1%)	72 (4.6%)	
**Estrogen, *n* (%)**				
−	24 (2.0%)	8 (2.1%)	32 (2.0%)	0.96^a^
+	1161 (98.0%)	380 (97.9%)	1541(98.0%)	
Missing	0	0	0	
**Progesterone, *n* (%)**				
−	135 (11.5%)	42 (11.0%)	177 (11.8%)	0.95^a^
+	1041 (88.5%)	346 (89.0%)	1385 (88.1%)	
Missing	9	2	11 (0.7%)	
**HER2, *n* (%)**				
−	1185 (100.0%)	0 (0.0%)	1185 (75.3%)	–
+	0 (0.0%)	388 (100.0%)	388 (24.7%)	
Missing	0	0	0	
**Ki67**				
Missing, n	868 (73.4%)	279 (97.6%)	1147 (72.9%)	< 0.05 ^a^
Mean (SD)	23.3 (19.2)	26.1 (17.9)	24.7 (18.9)	
Inclusion criterion^c^	***1072 (75.6%)***	***346 (24.4%)***	***1418 (100%)***	
**Adherence**				
No: ≤ 80%	146 (13.6%)	53 (15.4%)	199 (14.0%)	0.47 ^a^
Yes: > 80%	810 (75.5%)	259 (75.1%)	1069 (75.4%)	
Data not available^***d***^	117 (10.9%)	33 (9.6%)	150 (10.6%)	
**Patients with no missing**				
Data (N)^*e*^	955 (75.3%)	313 (24.7%)	1268 (100.0%)	
**Deceased (%)**^***f***^				
5-year, all causes (N, %)	96 (10.1%)	32 (10.1%)	153 (10.7%)	0.68 ^a^
10-year, all causes (N, %)	173 (18.1%)	57 (19.3%)	230 (18.1%)	
Years follow-up (mean, SD) ^g^	9.3 (1.8)	9.4 (1.7)	9.3 (1.8)	0.91 ^b^

^a^chi-square test, ^b^t-test, ^c^Inclusion criterion: patients diagnosed in stages I, II or III, ^d^Patients with no data available on adherence to endocrine treatment among patients who met the inclusion criterion *n* = 1418, ^e^Patients with no missing data among those who met the inclusion criterion who were included in the statistical modeling, ^f^number of deaths at 5 and 10 years of follow-up among the *N* = 1268 patients included in the modeling, ^g^follow-up of the *N* = 1268 patients included in the modeling.

Significant values are in bolditalics.

### All-cause survival analysis

**Figure 1:**
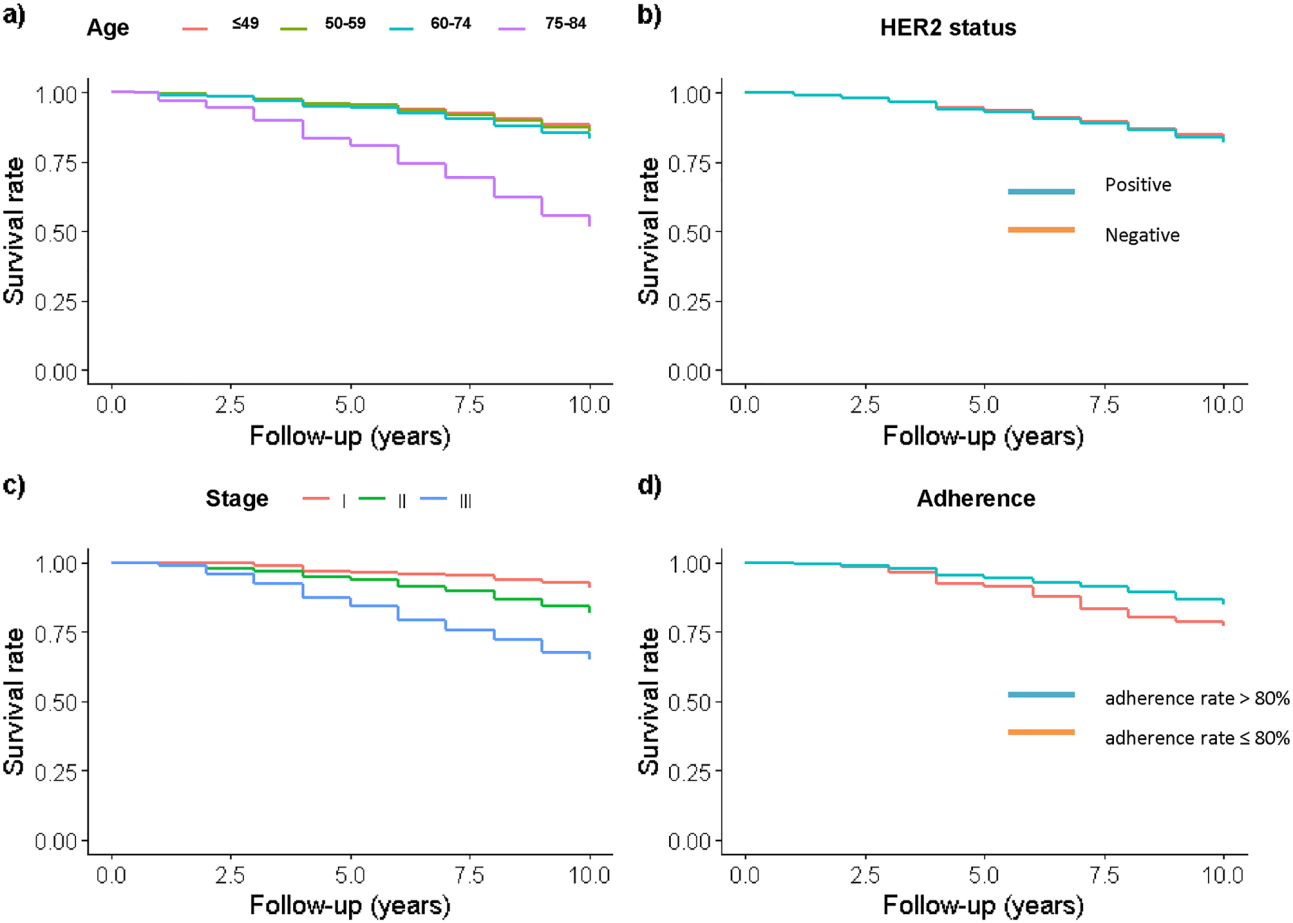
Patients diagnosed with hormone receptor-positive breast cancer before the age of 85 years in Girona and Tarragona: age-adjusted survival curves according to (**a**) age groups, (**b**) HER2 status, (**c**) stage at diagnosis, and (**d**) adherence to endocrine treatment.

Figure 1 depicts the cohort’s age-adjusted OS to 10 years according to age, molecular subtype, stage and ET adherence. Results show that (i) patients aged 75 years or more at diagnosis presented poorer survival (Panel a); (ii) survival did not show differences according to HER2 status (Panel b); (iii) survival decreased with stage at diagnosis (Panel c); (iv) at five years, survival was lower in non-adherent compared to adherent patients (Panel d).

### Cox modeling of λo(T)

Table 2 shows the adjusted hazard ratios and their corresponding 95% confidence intervals (CI) for the four Cox models considered. Compared to patients aged under 50, model C.1 showed about double the risk of death in those aged 60 to 74 (hazard ratio 2.08, 95% CI: 1.37–3.18) and a six-fold increase in those aged 75 to 84 (hazard ratio 6.14, 95% CI 4.08–9.25). Patients aged 50 to 59 years at diagnosis did not show a significantly increased risk. Risk of death was higher in patients diagnosed with stage II (hazard ratio 2.25, 95% CI 1.54–3.28) and stage III disease (hazard ratio 5.41, 95% CI 3.67–7.92) compared to patients in stage I. Patients adherent to ET showed a 42% lower risk of death (hazard ratio 0.58, 95% CI 0.42–0.82) than non-adherent patients.

**Table 2:** Hazard ratios derived from the four predictive models fitted: model C.1 considers age as categorical variable; model C.2 considers age as linear continuous variable; model C.3 considers restricted cubic splines on age; model C.4 considers restricted cubic splines on age and adherence as time varying.

	C.1 (age: categorical)		C.2 (age: linear)		C.3 (age: splines)		C.4 (age: splines/adherence: time-varying)	
	Hazard ratio	95% CI	Hazard ratio	95% CI	Hazard ratio	95% CI	Hazard ratio	95% CI
**Age**								
≤ 49 years	Ref		–		–		–	
50–59 years	1.44	0.91–2.88	–		–		–	
60–74 years	2.08	1.37–3.18	–		–		–	
75–84 years	6.14	4.08–9.25	–		–		–	
**HER2 status**								
Negative	Ref		Ref		Ref		Ref	
Positive	0.95	0.69–1.30	0.97	0.71–1.34	0.97	0.71–1.33	0.98	0.71–1.34
**Stage**								
I	Ref		Ref		Ref		Ref	
II	2.25	1.54–3.28	2.43	1.67–3.52	2.24	1.51–3.30	2.22	1.53–3.21
III	5.41	3.67–7.92	5.71	3.91–8.34	5.11	3.46–7.51	4.98	3.38–7.32
**Adherence rate**								
≤ 80%	Ref		Ref		Ref		–	
> 80%	0.58	0.42–0.82	0.54	0.39–0.76	0.57	0.41–0.79	–	
**Adherence (time varying)**								
Yes, follow-up ≥ 5 years	–		–		–		0.57	0.38–0.84
Yes, follow-up < 5 years	–		–		–		0.58	0.33–0.87
Ratio follow-up ≤ 5/ follow-up > 5***	–		–		–		1.02	0.51–2.02
AIC	2759.12		2753.25		2747.59		2759.54	

*CI* confidence interval, *AIC* Akaike Information Criterion, *: Beta coefficient for adherence was considered as time varying coefficient. **: hazard ratio for adherence for patients with follow-up > 5 years; *** Ratio of the hazard rates of the variable adherence = “Yes” for patients with follow-up ≤ 5 years versus > 5 years.

**Figure 2:**
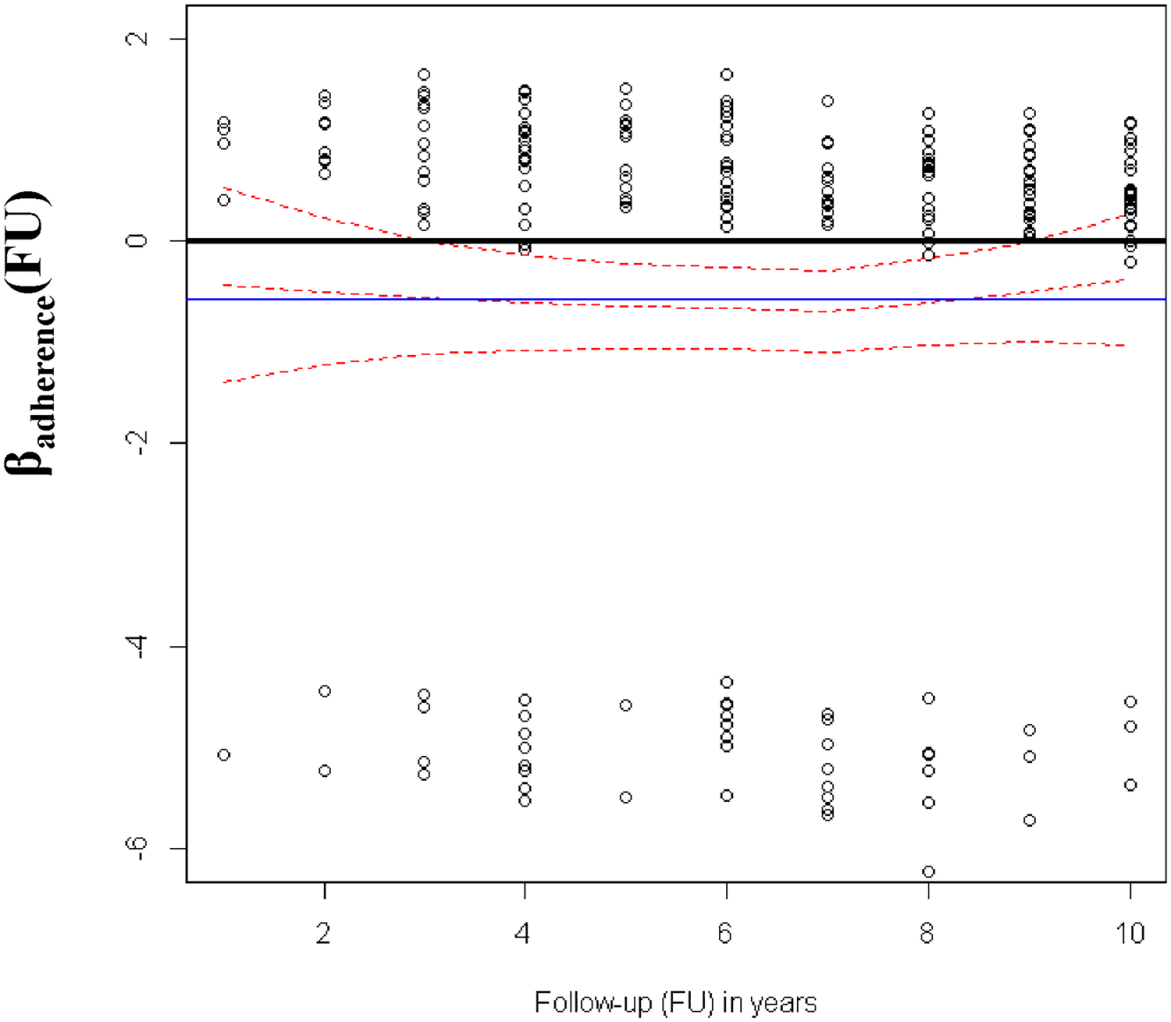
Plot of the time-dependent effect of “adherence” with 95% confidence intervals: in blue, log-hazard ratio coefficient, β_adherence_, estimated from the model with splines (model C.3) considering adherence as constant effect; in red, “time-varying” effect of “adherence” estimated through LOWESS regression of the scaled Schoenfeld residuals from model C.3 versus time (follow-up).

When age was considered as a continuous variable, hazard ratios for stage and adherence were similar (see models C.2, C.3, C.4), whereas the AIC decreased from 2759.12 in model C.1 to 2747.59 in model C.3. Figure 2 shows the plot for assessing the time-dependent effect of “adherence” with its 95% CI. The plot presents in blue the log-hazard ratio (horizontal line at β =  − 0.56; note that exp(− 056) = 0.57) derived from C.3, and in red the LOWESS regression of the scaled Schoenfeld residuals with respect to adherence coefficient in model C.3 versus time (follow-up). There is a slight curvature between follow-up years 1 and 4 and between 9 and 10; however, the 95% CI overlaps with the constant log-hazard ratio of β = –0.56. Moreover, we fitted a Cox model (C.4) with a time-varying coefficient for adherence considering 5 years as temporal cut-off (Table 2), observing no statistically significant differences between the two temporal periods (≤ 5 years and > 5 years), since the ratio of adherence coefficients between the two was 1.02 (95% CI 0.51–2.02) (see also Table 2 model C.4: hazard ratio of adherence for patient’s follow-up ≥ 5 years: 0.57; 95% CI 0.38–0.84; hazard ratio of adherence for patient’s follow-up < 5 years: 0.58; 95% CI 0.33–0.87). Therefore, we used model C.3 for predicting OS instead of model C.4, since it has fewer parameters and higher out-of-sample predictive features (AIC in model C.4: 2759.54; AIC model C.3: 2747.59). The graphical validation of the predictive performance of C.3 and the importance of the risk factors, depicted through a Nomogram, appears in the Supplementary Figs. S.1 and S.2.

### Differences in the P_BC_ comparing adherent versus non adherent patients

**Figure 3:**
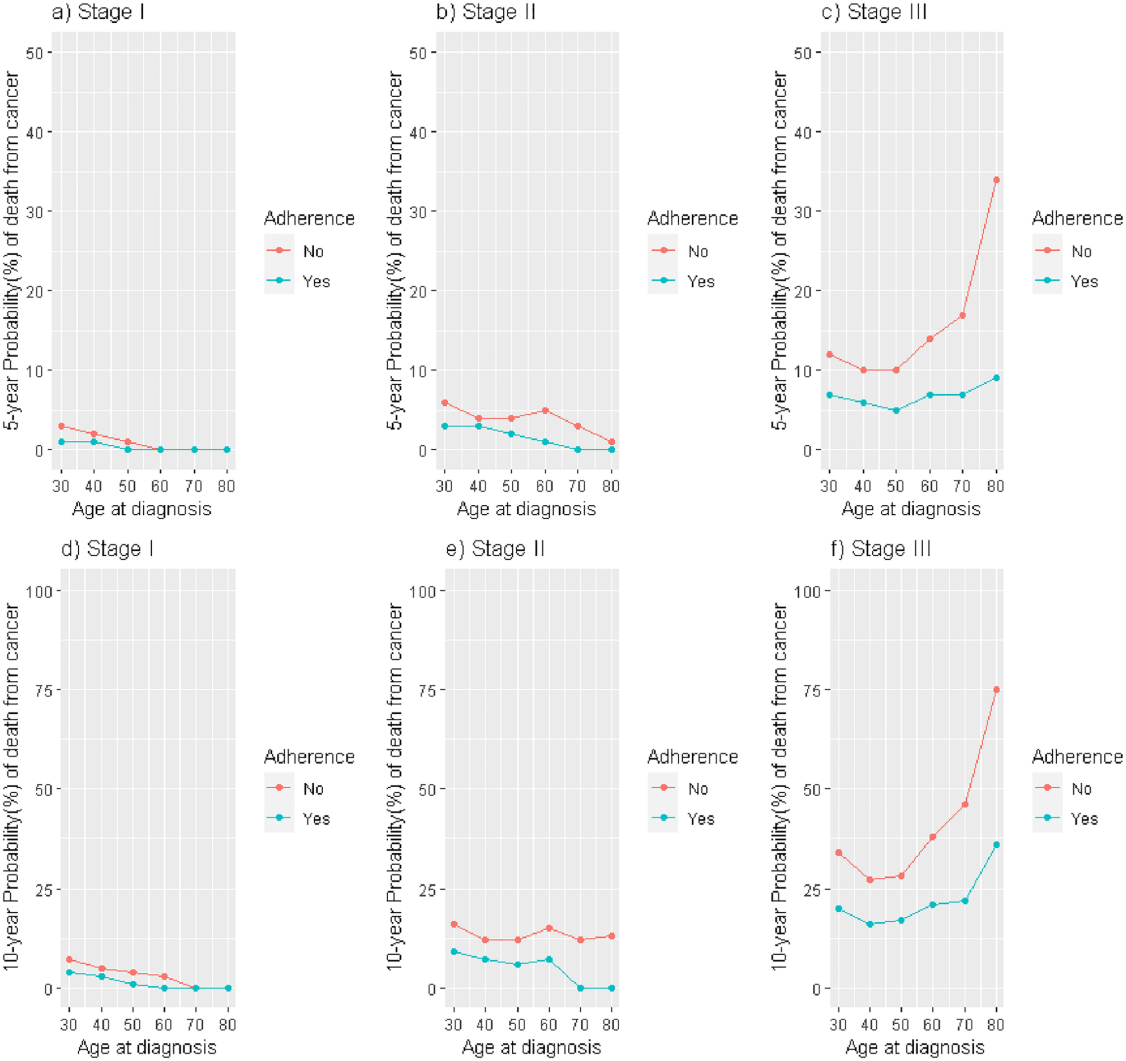
Predicted cumulative probabilities of death due to cancer at 5 (panels **a**–**c**) and 10 years (panels **d**–**f**) after diagnosis according to age and adherence to treatment (results presented by stage of BC at diagnosis).

Table 3 presents the 10-year cumulative P_BC_, comparing adherent versus non-adherent patients, aggregating all age groups and across all stages. Differences in P_BC_ between groups increased with stage, from 6.61% in stage I (95% CI 0.05–13.20) to 22.31% (95% CI 6.34–38.45) in stage III. Making use of λo(T) estimates from C.3, age-specific OS was predicted, and based on this, the age-trend of the 5-year and 10-year P_BC_ according to stage and adherence. Figure 3 shows differences in the age-trend of P_BC_ between adherent and non-adherent patients. In stage I, these differences were larger in patients diagnosed before 60 years of age (Fig. 3, Panels a and d), but in stage II, the differences in P_BC_(10) increased after age 50 (Fig. 3, Panel e). In stage III, the differences were even larger, showing a dramatic increase in P_BC_(5) in non-adherent patients aged over 50 (Fig. 3, Panel c), and an exponential rise for P_BC_(10) in patients diagnosed after age 60 (Fig. 3, Panel f).

**Table 3:** Ten-year cumulative crude probabilities of death due to breast cancer in the cohort, according to stage at diagnosis.

Stage	Probability of death due to breast cancer (%)				
	Adherent		Non-adherent		Difference
	*(N)*	P_BC_ (95% CI)	*(N)*	P_BC_ (95% CI)	P_BC_ (Non-adherent)—P_BC_ (Adherent) (95% CI)
I	461	2.20 (1.11–3.73)	75	8.81 (3.72–15.21)	6.61 (0.05; 13.20)
II	432	5.34 (2.81–9.32)	73	15.11 (8.20–23.93)	9.77 (0.59; 19.01)
III	176	20.10 (12.81–27.83)	51	42.41 (28.3–52.27)	22.31 (6.34; 38.45)

*P*_*BC*_ Probability of death due to Breast Cancer; 95% CI: 95% Confidence Interval.

Differences in the age-trend of the all-cause mortality between adherent and non-adherent patients were also assessed (see Fig. S3 and Table S.2, Supplementary material), showing marked differences between adherent and non-adherent patients in 10-year mortality across all stages, in a similar line to what was found for the P_BC_.

### BreCanSurvPred: use and interpretation of results

Once the end user accesses BreCanSurvPred, they select the age, stage and molecular subtype values, and then click on “Calculate Survival Indicators” to obtain results. Adherence (Yes/No) is available, but the user can compare differences in overall survival by selecting “Total”.

**Figure 4:**
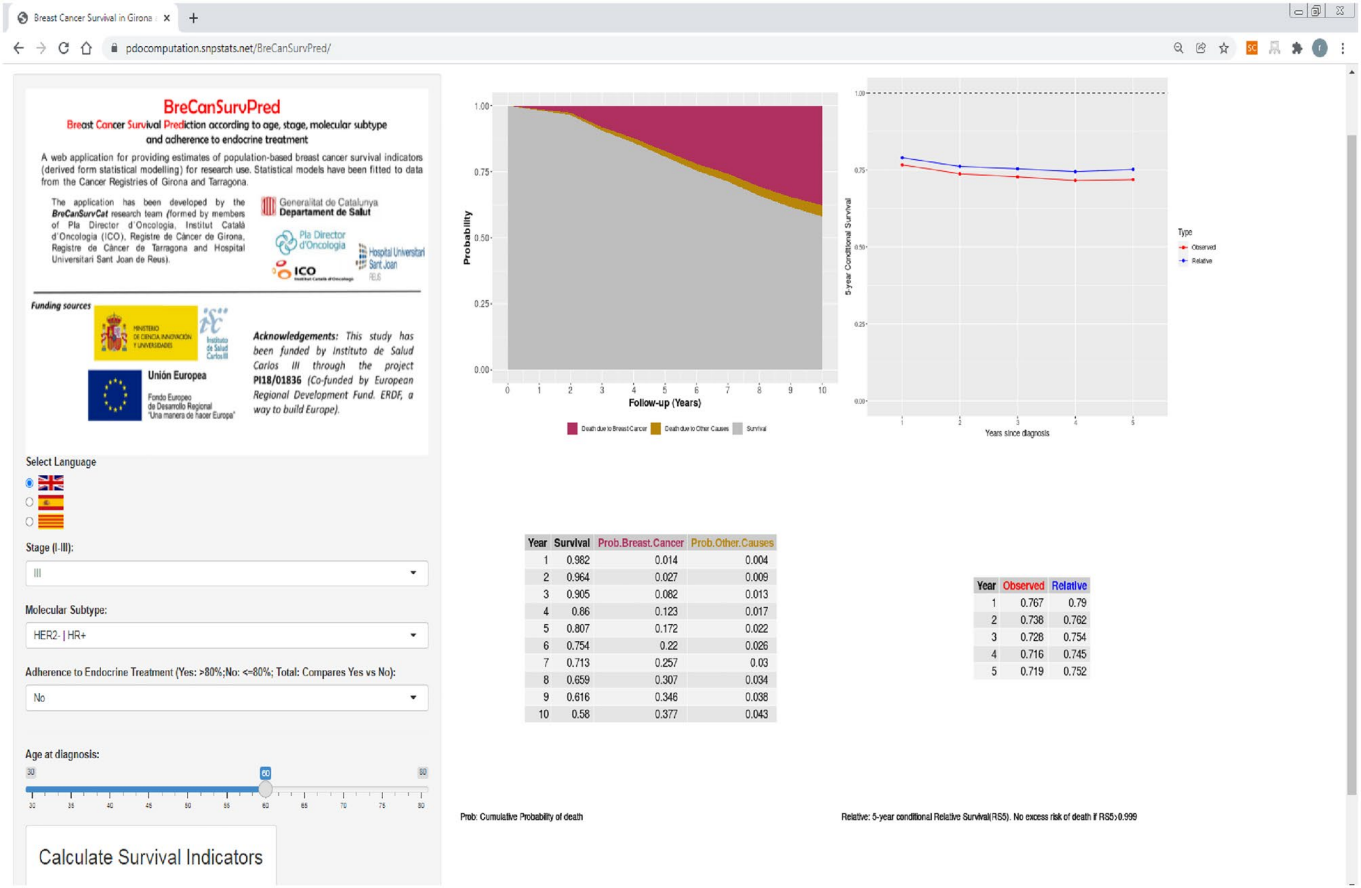
Snapshot of the web-based survival prediction application BreCanSurvPred. This snapshot demonstrates the probabilities of survival and death as well as the 5-year conditional probabilities of observed survival and relative survival for a 60-year old patient who was *not* adherent to endocrine therapy and who was diagnosed with stage III molecular subtype HER2 − /HR + breast cancer. These probabilites are calculated up to 10 years after BC diagnosis.

**Figure 5:**
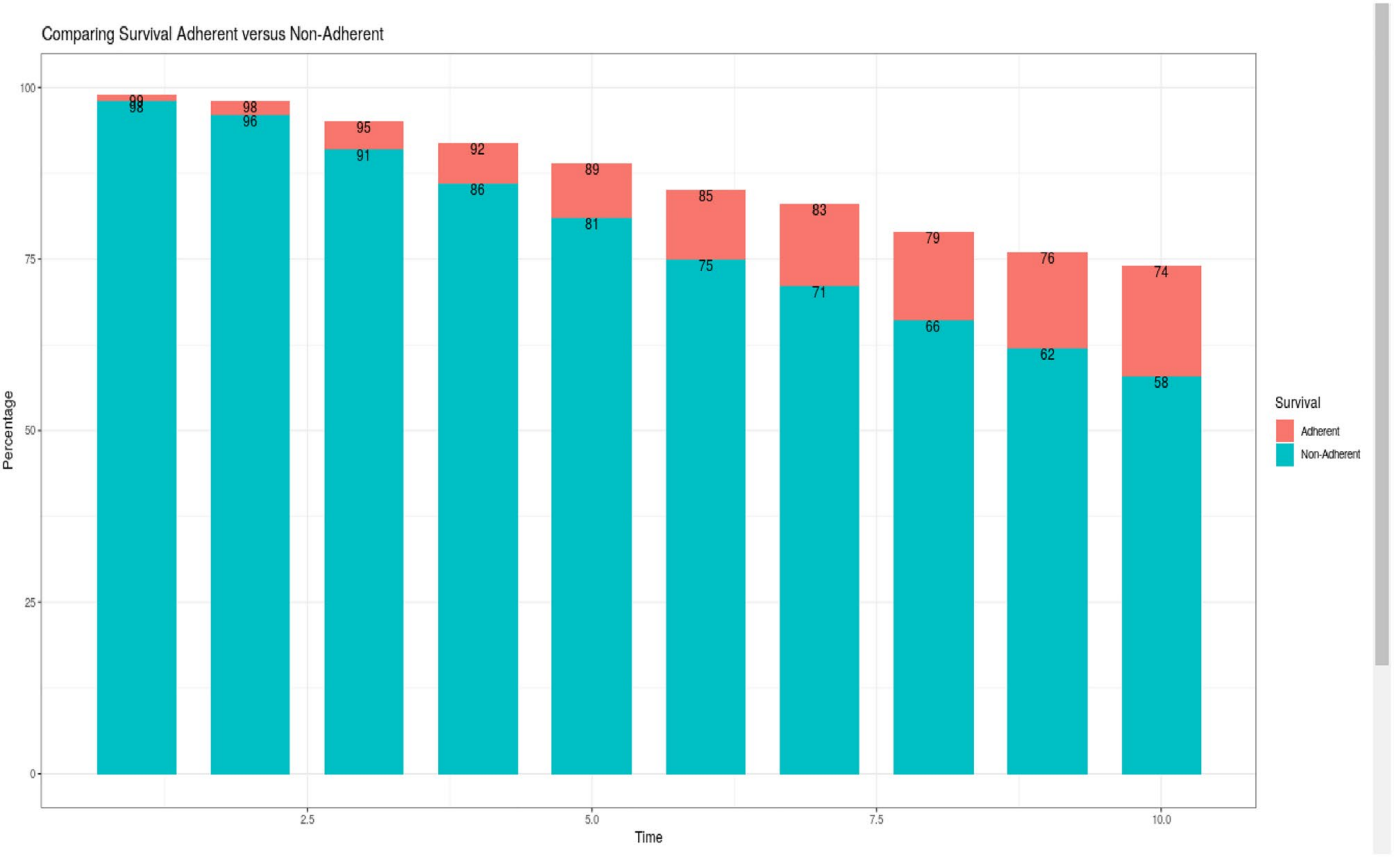
Snapshot of the web-based survival prediction application BreCanSurvPred. This snapshot shows the comparison of survival according to adherence to endocrine treatment for a 60-year old patient diagnosed with molecular subtype HER2 − /HR + in stage III. These survival probabilites are calculated up to 10 years after BC diagnosis. (Time: time since diagnosis [follow-up]; Percentage: percent survival at a certain Time ).

Figure 4 depicts the use of the web-application BreCanSurvPred for a 60-year-old, non-adherent patient diagnosed with a molecular subtype HER2 − /HR + breast cancer in stage III. For that patient, the model predicted an OS(10) of 58%, a P_BC_(10) of 37.7% and P_OC_(10) of 4.3%. The RS5 decreased slightly for the first five years after BC diagnosis: from RS5(1) 79% to RS5(5) 75.2%. Comparing this patient with the general population, the five-year excess risk of death was 25% (100 – RS5(5) = 24.8), which was conditional on already having survived five years after BC diagnosis. However, if that patient was adherent (Fig. S4, Supplementary material), her survival indicators would change significantly: the OS(10) could reach 73.5% and the P_BC_(10) could decrease to 21%. In this line, RS5(T) would be above 86% for all Ts. Therefore, comparing the indicators of that patient to those of a non-adherent one with similar characteristics, there would be a 1.27-fold increase in the OS (1.27 = 73.5/58), and a 45% reduction in the cumulative risk of death due to BC at 10 years from BC diagnosis (21/37.7 = 0.55, 1 − 0.55 = 0.45, 45% reduction). In this line, comparing this patient’s risk of death with that of the general population, the predicted EM was under 14% at five years after BC diagnosis, which is less than the 24.8% predicted for a non-adherent patient. Finally, a bar graph with a simple comparison of survival between adherent versus non-adherent patients can be obtained by selecting the option “Total: Compare Yes vs No” (Fig. 5). This bar graph can be used for discussing patients’ predicted annual survival based on their level of adherence to ET.

## Discussion

We have developed a web-based application to help oncologists talk with BC patients about their predicted survival up to 10 years according to age, stage, molecular subtype, and adherence to ET. Our modeling results show that patients with HR + BC and an ET adherence rate less than 80% for the first five years after BC diagnosis have higher P_BC_ than those with an adherence rate of 80% or more: + 6.6% in people diagnosed at stage I, + 9.77% at stage II, and + 22.3% at stage III. Adherence to ET when prescribed in HR + BC cases greatly reduces the risk of death due to BC at all stages, with some variation according to age at diagnosis. This fact could be attributable to the lower use and benefit of adjuvant chemotherapy in elderly people with BC.

A previous study using data from our cohort showed that non-adherence doubles the risk of death and increases the risk of recurrence 1.7-fold, after adjusting for age, registry, and year of diagnosis^18^. Evidence also shows that patients with low adherence rates to ET have an increased overall mortality risk^7,8,16,18,19,41–43^. Longer periods of adherence to ET at rates above 80% might lower the risk of death to below 30%^43^. We used the same rate as the cutoff value in our cohort to define adherence; however, other studies in BC survivors have used different threshold values^43^. Non-adherence, or waning adherence to ET over time present challenges for BC survival^43^, warranting intervention by the attending medical oncologist or multidisciplinary care team.

Our results are concordant with recent studies using data from a large SEER cohort comparing survival in different BC stubytpes^6^. In that study, differences in survival between HER2 + and HER2 − patients were minimal if these patients were also HR + , and the authors concluded that similar survival might be explained by the benefit of ET and HER2-targeted therapy for the HR + /HER2 + subtype^6,8^. We found a J-shaped trend between age and P_BC_ in stage III, with a flat trend for P_BC_ in the 40–49-year age group and increasing thereafter. The higher P_BC_ mortality before menopause could be explained by a more aggressive tumor biology and higher hereditary risk due to BRCA1 mutations^8,44^. Second, patients with HR + tumors might be at higher risk of relapse if they receive only chemotherapy^7,8^. Even with ET, primary and secondary tamoxifen resistance could lead to a worse prognosis for young patients with HR + BC^7,8,36^. Many strategies have been developed to improve the outcome of HR + BC, including ovarian function suppression in high-risk premenopausal patients and administration of ET beyond five years^7,8,16,27^. Recent clinical trials have also shed light on the role of cyclin inhibitors in early BC^8,27^. Given this complex scenario, prognostic tools can be useful in choosing the appropriate treatment for BC patients.

Evidence suggests that suboptimal medication adherence and discontinuation of treatment pose significant concerns for the management of BC patients^18–20,26–34^ with regard to disease recurrence^18–20,26,27,31,34^, risk of distant metastasis^26,29,30^, and/or increased mortality risk^18,26,28,30–34^. Adherence rates might show large variability, from 47 to 95%, depending on the age-group considered in the study^44^, or even lower rates in young premenopausal patients^26,45,46^. Postmenopause, an ET adherence rate of less than 90% has been associated with reduced disease-free survival^47^. A recent population-based study in Canada showed that the small proportion of postmenopausal BC patients who are non-adherent to a five-year course of ET^26^ may have concerns due to side effects/toxicity and perceive less benefit from ET^26,44^. For selected premenopausal patients, studies of extended aromatase inhibitors have yet to demonstrate benefits in overall survival^44^. In this line, during the recent 2021 St Gallen International Breast Cancer Conference, more than 80% of the participating experts supported considering extended ET for premenopausal patients with node-positive ER-positive HER2 − breast cancer^48^. Future research is warranted to explore optimal extended duration ET in this setting. On the other hand, the risk of undertreatment may increase with age^6–8^, probably due to comorbidities^44^. Older patients are less likely to receive surgery, chemotherapy, adjuvant radiotherapy, and ET^37^, and they may also present more side effects than younger patients, with the result that they show an increased P_BC_ at advanced stages^6–8,49–52^.

One of the strengths of this study is the population-based nature of cancer registry data. This minimizes the selection bias inherent to clinical trials, allows for complete ascertainment of incident BC cases (where information on stage and subtype was collected routinely), and enables the collection of follow-up data for up to 10 years after BC diagnosis. Another study’s strength is the collection and assessment of ET adherence data using a reliable and validated method for population cancer-registries^18,19^.

On the other hand, the data currently available in our study about prescription refill could be only calculated as cumulative “dose” at five years (or earlier if the patient died). This limits our analysis since patients’ adherence could change during the course of cancer treatment^44^. Although we did not find a time-dependent effect on the adherence coefficient, in a future study we must consider collecting this variable in time intervals in order to more precisely assess its relationship with mortality risk. In addition, we could assess adherence trajectories, which are related with predicting all-cause mortality^34,44^. Our aim was to develop a manageable web-based application to serve as a basic predictive tool, helping clinical oncologists to illustrate to patients the importance of actively adhering to ET. Thus, some key prognostic variables were not included, such as surgery, adjuvant treatment, tumor grade and comorbidities^3^. However, this is a major limitation for the use of the proposed nomograms when these data are available, as such variables have proven useful for predicting survival as well as the probability of death for up to five years after BC diagnosis^7,8,52–57^. Another limitation is that patients with incomplete clinical information for any of the four variables were excluded from the constructed nomograms. These nomograms are limited by the single data source and retrospective nature, so they should be validated using external cohorts. Moreover, even though the data are population-based, the size of the cohort is relatively small compared to other cohorts such as the SEER. Therefore, statistically significant differences between HER2-status through the hazard ratios could probably not be detected. Finally, our study estimated the P_BC_ because cause-specific mortality was not available. While P_BC_ is a reliable and comparable indicator between population-based studies^22–25^, the combined analysis of cause-specific and P_BC_ could show a more realistic scenario of the “real” risk of death due to BC and other causes^25^.

Finally, the inclusion of adherence to ET—combined with stage information and BC subtype—in population-based survival studies must be recommended since it improves the explanation of the favorable evolution of survival trends. Recent studies in Spain showed high five-year survival rates, above 90%, thanks in part to the increased detection of BC at early stages^58^. The success of the screening programs, together with introduction of effective treatments, plays a significant role in the decreasing BC mortality risk in Spain^59^. However, we have also shown that survival differs significantly within stage when stratified by treatment. Moreover, differences in age-adjusted survival between Spain and other industrialized countries are modest^60^, and the use of P_BC_ and P_OC_ must be accounted for when comparing reliable probabilities of death between countries^25^.

In conclusion, our results suggest that: (i) stage and age are the most important factors in predicting the risk of death due to BC among patients with HR + tumors; (ii) in patients who are prescribed ET, adherence for the first five years after BC diagnosis has a major impact on 10-year overall survival and P_BC_ in stages I-III. The BreCanSurvPred application graphically depicts the progression of the survival, P_BC_ and P_OC_ curves, which could be useful for illustrating to patients the consequences of non-adherence to ET when prescribed.

## Supplementary Information

Supplementary Information.

## Data Availability

Data will be provided on reasonable request.

## Abbreviations

BC: Breast cancer
IHC: Immunohistochemistry
HR: Hormone receptors
HER2: Human epidermal growth factor receptor
ET: Endocrine therapy
HER2E: Her2-enriched
TN: Triple Negative
OS(T): Observed survival at time T
P_BC_(T): The probability of death due to BC
P_OC_(T): The probability of death due to other causes
OS5(T): Five-year observed conditional survival
RS5(T): Five-year conditional relative survival
EM(T): Excess mortality, expressed as percentage, EM(T) = [1 − RS5(T)] × 100.
AIC: Akaike Information Criterion
CI: Confidence Interval
