# Supplementary material for: Using population-based data to evaluate the impact of adherence to endocrine therapy on survival in breast cancer through the web-application BreCanSurvPred
Source: Sci Rep. 2022 May 16;12:8097. doi: 10.1038/s41598-022-12228-y (PMC9110408; doi:10.1038/s41598-022-12228-y)
Supplement: Supplementary file 1 — Supplementary Information. [file 41598_2022_12228_MOESM1_ESM.docx]

***Supplementary material for:***

**“Using population-based data to evaluate the impact of adherence to endocrine therapy on survival in breast cancer through the web-application BreCanSurvPred”**

Rebeca Font^1,2,+^, Maria Buxó^3,+^, Alberto Ameijide^4^, José Miguel Martínez^5,6^, Rafael Marcos-Gragera^7,8,9^, Marià Carulla^4^, Montse Puigdemont^7^, Mireia Vilardell^10^, Sergi Civit^11^, Gema Viñas^12^, Josep A. Espinàs^1,2^, Jaume Galceran^4^, Ángel Izquierdo^7,12^, Josep M. Borràs^1,2,13^, Ramon Clèries^1,2,13,*^.

1. Pla Director d’Oncología. Av Gran Vía 199-203 08908 Hospitalet de Llobregat;
2. Institut d’Investigació Biomèdica de Bellvitge, IDIBELL. Av. Gran Via de l’Hospitalet, 199-203 – 1a planta. 08908 Hospitalet de Llobregat(Barcelona), Spain.
3. Institut d’Investigació Biomèdica de Girona, IDIBGI. C/Dr.Castany s/n. Edifici M2. Parc Hospitalari Martí i Julià. 17190 Salt, Spain.
4. Registre de Càncer de Tarragona, Servei d'Epidemiologia i Prevenció del Càncer, Hospital Universitari Sant Joan de Reus, IISPV, Reus, Spain.
5. Department de Estadística i Investigació Operativa de la Universitat Politècnica de Catalunya. EDIFICI H. Diagonal 647, 08028 Barcelona, Spain
6. Grupo de Investigación en Salud Pública, Universidad de Alicante, 03690 Alicante, Spain.
7. Registre de Cáncer de Girona – Unitat d’Epidemiologia. Pla Director d’Oncologia. Institut Català d’Oncología. Grup d’Epidemiologia Descriptiva, Genètica i Prevenció del Càncer de Girona-IDIBGI, Girona 17005.
8. Facultat de Medicina, Universitat de Girona (UdG), Girona, Spain.
9. Centro de Investigación Biomédica en Red: Epidemiología y Salud Pública (CIBERESP), Madrid, Spain.
10. Independent Researcher
11. Secció de Estadística del Departament de Genètica, Microbiología i Estadística de la Facultat de Biologia. Universitat de Barcelona 08028.
12. Servei d’Oncología Médica. Institut Català d’Oncología. Hospital Universitari de Girona Doctor Josep Trueta, Girona 17005.
13. Department de Ciències Clíniques de la Universitat de Barcelona 08907

***^+^Equal contribution***

^*^ ***Corresponding author:*** Ramon Clèries, PhD**.** Pla Director d’Oncología. IDIBELL, Av Gran Vía 199-203 08908 Hospitalet de Llobregat e-mail: [r.cleries@iconcologia.net](mailto:r.cleries@iconcologia.net)

***Index of Tables and Figures***

**Page 3. Table S1.1.** Characteristics of the whole cohort of patients diagnosed with breast cancer before the age of 85 years in Girona and Tarragona (Spain), 2007 to 2009

**Page 4. Table S1.2** Characteristics of patients diagnosed with hormone receptor-positive breast cancer before the age of 85 years in Girona and Tarragona, 2007 to 2009

**Page 10 Figure S1.** Calibration plots of the Cox models fitted to all data using age as a categorical variable and restricted cubic splines on age with knots at 25, 49, 59, 74 and 84 years: Panel (a) assessment for predicting 5-year survival; Panel (b) assessment for predicting 10-year survival.

**Page 11. Figure S2.** Nomograms derived from fitting a Cox model to the dataset with patients eligible for endocrine therapy and with information available on adherence to ET during their 5 five years after BC diagnosis (patients diagnosed in stages I, II or III with HER2-positive or HER2-negative).

**Page 11. Figure S3.** Predicted cumulative probabilities of all-cause mortality at 5 (panels a-c) and 10 years (panels d-f) after BC diagnosis according to age and adherence to treatment (results presented by stage of BC at diagnosis).

**Page 12. Table S2.** Ten-year cumulative crude probabilities of death in the cohort, according to stage at diagnosis

**Page 14. Figure S4.** Snapshot of the web-based survival prediction application BreCanSurvPred. This snapshot demonstrates the probabilities of survival and death as well as the 5-year conditional probabilities of observed survival and relative survival for a 60-year-old patient who was *adherent* to ET and diagnosed with molecular subtype HER2- | HR + in stage III. These probabilities are calculated up to 10 years after BC diagnosis.

**Table S1.1** Characteristics of the whole cohort of patients diagnosed with breast cancer before the age of 85 years in Girona and Tarragona (Spain), 2007 to 2009

|  | ***HER2−/HR+****  ***(N=1185)*** | ***HER2+/HR+****  ***(N=388)*** | ***HER2ENRICHED***  ***(N=137)*** | ***Triple negative***  ***(N=160)*** | | ***Missing***  ***(N=179)*** | | | ***Total***  ***(N=2049)*** | | |  |  |
| --- | --- | --- | --- | --- | --- | --- | --- | --- | --- | --- | --- | --- | --- |
| **Registry, n (%)** |  |  |  |  | | |  | | |  | | |  |
| Girona | 599 (50.5) | 208 (53.6) | 69 (50.4) | 90 (56.2) | | | 40 (22.3) | | | 1006 (49.1) | | |  |
| Tarragona | 586 (49.5) | 180 (46.4) | 68 (49.6) | 70 (43.8) | | | 139 (77.7) | | | 1043 (50.9) | | |  |
| **Age** |  |  |  |  | | |  | | |  | | |  |
| Mean (SD) | 58.9 (12.9) | 57.4 (13.2) | 55.8 (13.6) | 55.4 (13.1) | | | 63.1 (14.8) | | | 58.5 (13.4) | | |  |
| **Age groups, n (%)** |  |  |  |  | | |  | | |  | | |  |
| 0–49 years | 329 (27.8) | 121 (31.2) | 45 (32.8) | 57 (35.6) | | | 41 (22.9) | | | 593 (28.9) | | |  |
| 50–59 years | 291 (24.6) | 103 (26.5) | 44 (32.1) | 45 (28.1) | | | 35 (19.6) | | | 518 (25.3) | | |  |
| 60–74 years | 389 (32.8) | 113 (29.1) | 31 (22.6) | 41 (25.6) | | | 40 (22.3) | | | 614 (30.0) | | |  |
| 75–84 years | 176 (14.9) | 51 (13.1) | 17 (12.4) | 17 (10.6) | | | 63 (35.2) | | | 324 (15.8) | | |  |
| **Stage at diagnosis, n (%)** | | | | |  | | |  | | |  | | |
| I | 473 (39.9) | 116 (29.9) | 35 (25.5) | 41 (25.6) | | | 43 (24.0) | | | 708 (34.6) | | |  |
| II | 416 (35.1) | 160 (41.2) | 50 (36.5) | 67 (41.9) | | | 36 (20.1) | | | 729 (35.6) | | |  |
| III | 183 (15.4) | 70 (18.0) | 33 (24.1) | 32 (20.0) | | | 8 (4.5) | | | 326 (15.9) | | |  |
| IV | 57 (4.8) | 26 (6.7) | 16 (11.7) | 12 (7.5) | | | 14 (7.8) | | | 125 (6.1) | | |  |
| Missing | 56 (4.7) | 16 (4.1) | 3 (2.2) | 8 (5.0) | | | 78 (43.6) | | | 161 (7.9) | | |  |
| **Follow-up, years, mean (SD)** | 9.3 (1.8) | 9.4 (1.7) | 7.9 (2.9) | 7.8 (2.9) | | | 6.9 (2.9) | | | 9.0 (2.7) | | |  |

HR+: hormone receptor-positive

**Table S1.2** Characteristics of patients diagnosed with hormone receptor-positive breast cancer before the age of 85 years in Girona and Tarragona, 2007 to 2009

|  | ***HER2-***  ***(N=1185;75.3%)*** | ***HER2+***  ***(N=388;24.7%)*** | ***Total***  ***(N=1573; 100%)*** | ***p-value*** |
| --- | --- | --- | --- | --- |
| **Registry, n (%)** |  |  |  |  |
| Girona | 599 (50.5%) | 208 (53.6%) | 807 (51.3%) | 0.29^a^ |
| Tarragona | 586 (49.5%) | 180 (46.4%) | 766 (48.7%) |  |
| **Age** |  |  |  |  |
| Mean (SD) | 58.9 (12.9) | 57.4 (13.2) | 58.3 (13.1%) | 0.87 ^b^ |
| **Age groups, n (%)** |  |  |  |  |
| 0–49 years | 329 (27.8%) | 121 (31.2%) | 450 (28.6%) | 0.32 ^a^ |
| 50–59 years | 291 (24.6%) | 103 (26.5%) | 394 (25.1%) |  |
| 60–74 years | 389 (32.8%) | 113 (29.1%) | 502 (31.9%) |  |
| 75–84 years | 176 (14.9%) | 51 (13.1%) | 227 (14.4%) |  |
| **Stage** |  |  |  |  |
| I | 473 (39.9%) | 116 (29.9%) | 589 (37.4%) | <0.05 ^a^ |
| II | 416 (35.1%) | 160 (41.2%) | 576 (36.6%) |  |
| III | 183 (15.4%) | 70 (18.0%) | 253 (16.1%) |  |
| IV | 57 (4.8%) | 26 (6.7%) | 83 (5.3%) |  |
| Missing | 56 (4.7%) | 16 (4.1%) | 72 (4.6%) |  |
| **Estrogen, n (%)** |  |  |  |  |
| - | 24 (2.0%) | 8 (2.1%) | 32 (2.0%) | 0.96 ^a^ |
| + | 1161 (98.0%) | 380 (97.9%) | 1541(98.0%) |  |
| Missing | 0 | 0 | 0 |  |
| **Progesterone, n (%)** |  |  |  |  |
| - | 135 (11.5%) | 42 (11.0%) | 177 (11.8%) | 0.95 ^a^ |
| + | 1041 (88.5%) | 346 (89.0%) | 1385 (88.1%) |  |
| Missing | 9 | 2 | 11 (0.7%) |  |
| **HER2, n (%)** |  |  |  |  |
| - | 1185 (100.0%) | 0 (0.0%) | 1185 (75.3%) | - |
| + | 0 (0.0%) | 388 (100.0%) | 388 (24.7%) |  |
| Missing | 0 | 0 | 0 |  |
| **Ki67** |  |  |  |  |
| Missing, n | 868 (73.4%) | 279 (97.6%) | 1147 (72.9%) | <0.05 ^a^ |
| Mean (SD) | 23.3 (19.2) | 26.1 (17.9) | 24.7 (18.9) |  |
| ***Inclusion criterion ^c^*** | ***1072 (75.6%)*** | ***346 (24.4%)*** | ***1418 (100%)*** |  |
| **Adherence** |  |  |  |  |
| No: ≤ 80% | 146 (13.6%) | 53 (15.4%) | 199 (14.0%) | 0.47 ^a^ |
| Yes: >80% | 810 (75.5%) | 259 (75.1%) | 1069 (75.4%) |  |
| Data not available ***^d^*** | 117 (10.9%) | 33 (9.6%) | 150 (10.6%) |  |
| ***Patients with no missing***  **data (N) *^e^*** | 956 (75.3%) | 312 (24.7%) | 1268 (100.0%) |  |
| **Deceased (%) *^f^*** |  |  |  |  |
| 5-year, all causes (N, %) | 96 (10.1%) | 32 (10.1%) | 153 (10.7%) | 0.68 ^a^ |
| 10-year, all causes (N, %) | 173 (18.1%) | 57 (19.3%) | 230 (18.1%) |  |
| **Estrogen +, n (%) ^g^** | 956 ( 100%) | 312 ( 100%) | 1268 ( 100%) |  |
| **Years follow-up (mean, SD) ^h^** | 9.3 (1.8) | 9.4 (1.7) | 9.3 (1.8) | 0.91 ^b^ |

a: chi-square test; b: t-test;

c: Inclusion criterion: patients diagnosed in stages I, II or III; d: Patients with no data available on adherence to endocrine treatment among patients who met the inclusion criterion n=1418; e: Patients with no missing data among those who met the inclusion criterion who were included in the statistical modeling; f: number of deaths at 5 and 10 years of follow-up among the N=1268 patients included in the modeling; g: estrogen receptor positive among selected patients; h: follow-up of the N=1268 patients included in the modeling

***Extension of the statistical methods***

***Indicators used***

We calculated the observed survival (OS) up to 31 December 2019 by using the Kaplan-Meier method for assessing differences in survival according to the explanatory variables. These were included into a Cox multivariate regression model from which we derived the corresponding hazard ratios. Making use of competing risks modeling, which combines proportional hazards models for all-cause mortality and relative survival^24,25^, the P_BC_(T) and P_OC_(T) were calculated. Their analysis depends on the estimation of the λo(T), the overall hazard of death in the cohort at a specific time T, which can be obtained by means of a Cox model fitted to the cohort data^24^. The observed survival at any time T can be predicted as OS(T)=$\int_{0}^{T} exp[-\lambda_{O}\left( u \right)]du$^22-25^.

Under additive modeling, the excess hazard of death in the cohort due to BC is defined as λ_X_(T)=λ_O_(T)-λ_P_(T), where λ_P_(T) is the expected hazard of death in the cohort according to the general population’s mortality rates^24^. Here we used the all-cause mortality rates for 2007-2019 in Catalonia, the administrative region of Spain encompassing both cancer registries, to estimate ES. Using λ_X_(T) and λ_P_(T), it is possible to calculate the expected survival at time T, ES(T)=$\int_{0}^{T} exp[-\lambda_{P}\left( u \right)]du$ , and also P_BC_(T)=$\int_{0}^{T} \mathrm{OS}\left( u \right)\lambda_{X}\left( u \right)du$as well as P_OC_(T)$=\int_{0}^{T} \mathrm{OS}\left( u \right)\lambda_{P}\left( u \right)du.$ Finally, from the OS(T) and ES(T), we can estimate the RS(T)=OS(T)/ES(T), and from this quantity^24^, the five-year conditional relative survival (RS5), RS5(T)=RS(T+5)/RS(T)^25^. RS5(T) represents the patient’s five-year survival, conditional on having survived at least T years after BC diagnosis, compared to the expected survival of the general population-cohort of the same age during the period of diagnosis^25^. Moreover, [1− RS5(T)]×100 can be used to estimate the “excess mortality”, EM of the patients compared to the risk in the general population ^36,37^. Population-based studies focus on the EM(T) between one and five years, since values between 0% and 5% could indicate that the population may not present EM compared to the general population during that time interval^25,36,37^, a concept related with “cancer cure” if extrapolated beyond T>5 (that is > 10 years of follow-up)^37^. For example, RS5(3) is the five-year survival of the patient who has survived at least three years after BC diagnosis. An RS5(3) of 0.95 would mean that the patient will have a 5% excess risk of death due to cancer five years after the third year of follow-up. Moreover, if RS5(T) was 1 for a certain T, that indicates no excess risk of death due to cancer. These RS5 estimates can help clinicians make decisions, for instance with regard to adjusting or personalizing treatments and determining patients’ long-term prognosis.

The relative survival approach used here to estimate the crude probabilities of death due to cancer and due to other causes requires the general’s population mortality rates in order to estimate the expected survival rates. Since OS(T)+M(T)=1, where M(T) is the all-cause mortality, the RS approach allows partitioning M(T) into P_BC_(T) and P_OC_(T), such that M(T)= P_BC_(T)+P_OC_(T). This approach is using general’s population mortality for providing P_BC_(T) and P_OC_(T) estimates when cause-specific mortality in the cohort is not available, but it is not used for exchanging survival in the BC cohort with that of the general population.

***Cox modeling procedure***

The first step was to fit a Cox model to the study population’s dataset using λo(T) as the outcome. The impact of each one of the variables on λo(T) was assessed by deriving their HRs from that Cox model but considering the age variable as categorical, with the four categories described above, or alternatively as continuous. We assessed four Cox models with different assumptions according to age and adherence to endocrine therapy (ET): i) model C.1 (age as categorical); ii) model C.2 (age as log-linear continuous variable); iii) model C.3 (age as continuous using restricted cubic splines); and iv) model C.4 (model C.3 but also considering adherence as a time-varying variable). When considering restricted cubic splines, age was partitioned into windows (“pieces”) and within each window there was a cubic polynomial^38-40^. These windows are defined by “knots”, for which we used the cut-off age values 49 years, 59 years, and 74 years. The Akaike Information Criterion (AIC) was used to select the “candidate” model^39,40^. Evidence suggests that the use of restricted cubic splines on age adds flexibility to the predictive ability of the Cox model due to the potential non-linear relationship between age and λo(T)^24,40^. On the other hand, we assessed whether the coefficient related with adherence to ET must be considered as time dependent since the proportion of days covered is based on a denominator of five years, but this denominator changes if a person dies or recurs during the five years. Also, given the high dose of most endocrine therapies^7,8^, it is also necessary to assess whether significant temporal changes in the adherence coefficient take place in order to avoid person-time bias, which could be affected by early discontinuation over proportion of days covered. We used a locally weighted scatter-plot smoother, LOWESS, of the scaled Schoenfeld residuals versus a log-transformation of time^38^ to visualize the plausibility of considering a time-dependent effect for the Cox model’s adherence coefficient.

***Validation of the selected Cox model: variable importance and*** λo(T) ***predictions***

We used a Nomogram to assess the importance (“weight”)^39^ of the prognostic factors in predicting OS(5) and OS(10). The Cox model selected and used to develop that Nomogram was internally validated and calibrated by fitting the model up to 1000 times to a random sample including 60% of the original data (training dataset) and using the remaining 40% as validation data. The discrimination ability of the Cox model’s prediction was evaluated by Harrell’s concordance index (C-index), and the corresponding calibration curves were also derived to assess the correlation between the actual OS in the cohort and the predicted Cox model survival^39^.

***R-packages used***

We used the R-package survminer (<https://CRAN.R-project.org/package=survminer>) for calculating these age-adjusted curves. Next, we fitted four Cox models to the data to identify the model with the best fit. We also assessed temporal changes in Cox model coefficients, developed a Nomogram for depicting the impact of each variable, and internally validated this model through resampling techniques, using the R-packages survival (<https://CRAN.R-project.org/package=survival>) and rms (<https://cran.r-project.org/package=rms>). Crude probabilities of death were calculated by estimating observed and expected survival according to Ederer II method^24,25,29,31^, using the R-package relsurv (<https://CRAN.R-project.org/package=relsurv>). The web application for obtaining these indicators was developed through the R-package shiny (<https://CRAN.R-project.org/package=shiny>).

***Extension of Results***

***Predictive performance of the Cox models***.

Predictive performance of the Cox models.

First, we evaluated Harrell’s concordance index of subsequent partitions of the dataset into the training (760/1218, 60%) and validation ones (508/1218, 40%). We compared the performance of the “worst” AIC model, model C.1, with that with “best” AIC model, model C.3, both fitted to the training dataset using 1000 bootstrap resamples (N=760). A C-Index ranging from 0.63 to 0.75 was estimated for model C.1 and from 0.78 to 0.89 for model C.3 when both were applied to the validation dataset. A calibration plot verified that the predicted survival using these Cox models was strongly correlated with the actual OS (Supplementary material Fig. S1). Calibrations were performed for 5- and 10-year overall survival. Although there were no statistically significant differences in the predictions, model C.3 was selected since its AIC was smaller than that of model C.1.

The next step was to graphically visualize the impact of the variables in predicting the overall survival; for that purpose, the hazard ratios derived from model C.3 were integrated into a Nomogram. The value of each of these variables was given a score on the point scale axis, where the estimated probability of 5-year and 10-year OS could be calculated by counting the scores and locating them on the total point scale (Supplementary material Fig. S2). Based on this Nomogram, we found that stage and age were the variables providing the largest scores (up to 83 points for patients in stage III, and beyond 80 points for patients in the older age groups), followed by adherence, from which “non-adherent” patients scored 30 extra points in that Nomogram. We assessed the predictive performance in the Cox models by evaluating Harrell’s concordance index of subsequent partitions of the dataset into the training (760/1218, 60%) and validation ones (508/1218, 40%). We compared the performance of the Cox models fitted to the training dataset using 1000 bootstrap resamples, obtaining a C-Index ranging from 0.78 to 0.89 for the model C.3 (the selected one, with lowest AIC, see main text, Results section). A calibration plot verified that the predicted survival using these Cox models was strongly correlated with the actual OS (Fig. S1). Calibrations were performed for 5- and 10-year survival and with two Cox models, one that used age as a categorical variable and another that used restricted cubic splines. There were no statistically significant differences in the prediction, but using the model with age as a continuous variable allows for more flexibility in out-of-sample predictions.

***Importance of the risk factors for predicting BC survival***

To assess the importance of the prognostic factors, the HRs derived from Cox modeling were integrated into a Nomogram. The value of each of these variables was given a score on the point scale axis, where the estimated probability of 5- and 10-year OS was calculated by counting the scores and locating them on the total point scale (Fig. S2). Based on this Nomogram, we found that age and stage were the variables providing the largest scores, followed by adherence.

**Figure S1.** Calibration plots of the Cox models fitted to all data using age as a categorical variable and restricted cubic splines on age with knots at 25, 49, 59, 74 and 84 years: Panel (a) assessment for predicting 5-year survival; Panel (b) assessment for predicting 10-year survival.

**Figure S2.** Nomograms derived from fitting a Cox model to the dataset with patients eligible for endocrine therapy and with information available on adherence to endocrine therapy for 5 five years after BC diagnosis (patients diagnosed in stages I, II or III with HER2-positive or HER2-negative).


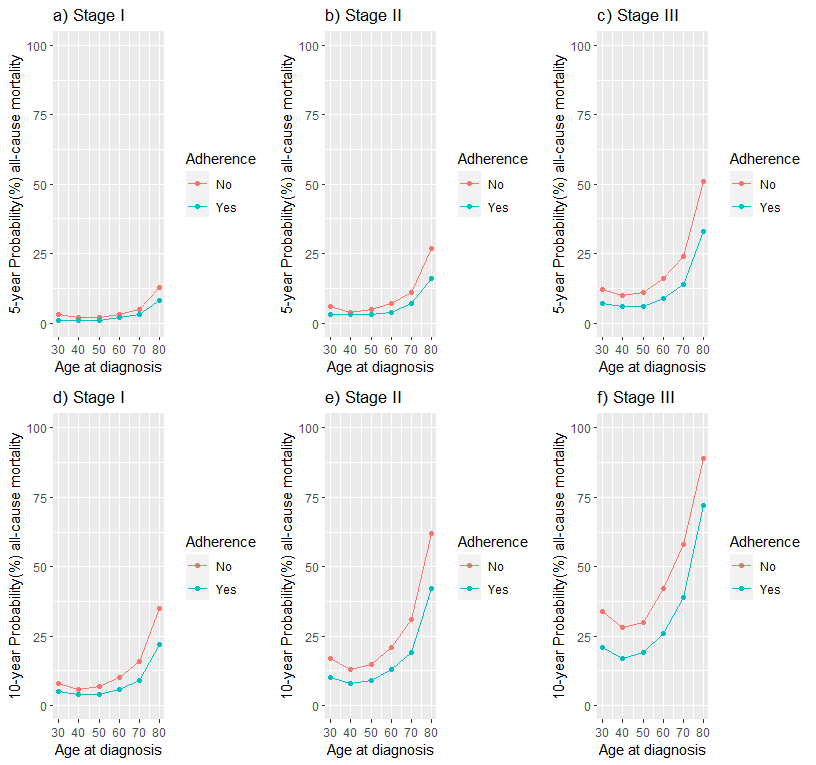


**Figure S3.** Predicted cumulative probabilities of all-cause mortality at 5 (panels a-c) and 10 years (panels d-f) after BC diagnosis according to age and adherence to treatment (results presented by stage of BC at diagnosis).

**Table S2.** Ten-year cumulative crude probabilities of death in the cohort, according to stage at diagnosis

|  | **Probability of death (%)** | | | | | |
| --- | --- | --- | --- | --- | --- | --- |
|  | *Adherent* | |  | *Non-adherent* | | *Difference* |
| *Stage* | *(N)* | P_D_ (95% CI) |  | *(N)* | P_D_ (95% CI) | P_D_ (Non-adherent) - P_D_ (Adherent) (95% CI) |
| I | 461 | 6.92 (4.60-9.20) |  | 75 | 12.91 (4.32-21.14) | 6.01 (0.01; 8.81) |
| II | 432 | 11.00 (9.32-20.41) |  | 73 | 19.93 (9.63-27.71) | 8.93 (0.01; 17.61) |
| III | 176 | 31.82 (24.62-38.41) |  | 51 | 45.11 (29.63-57.24) | 13.29 (0.03; 29.81) |

P_D_ : Probability of death in the cohort; 95% CI: 95% Confidence Interval


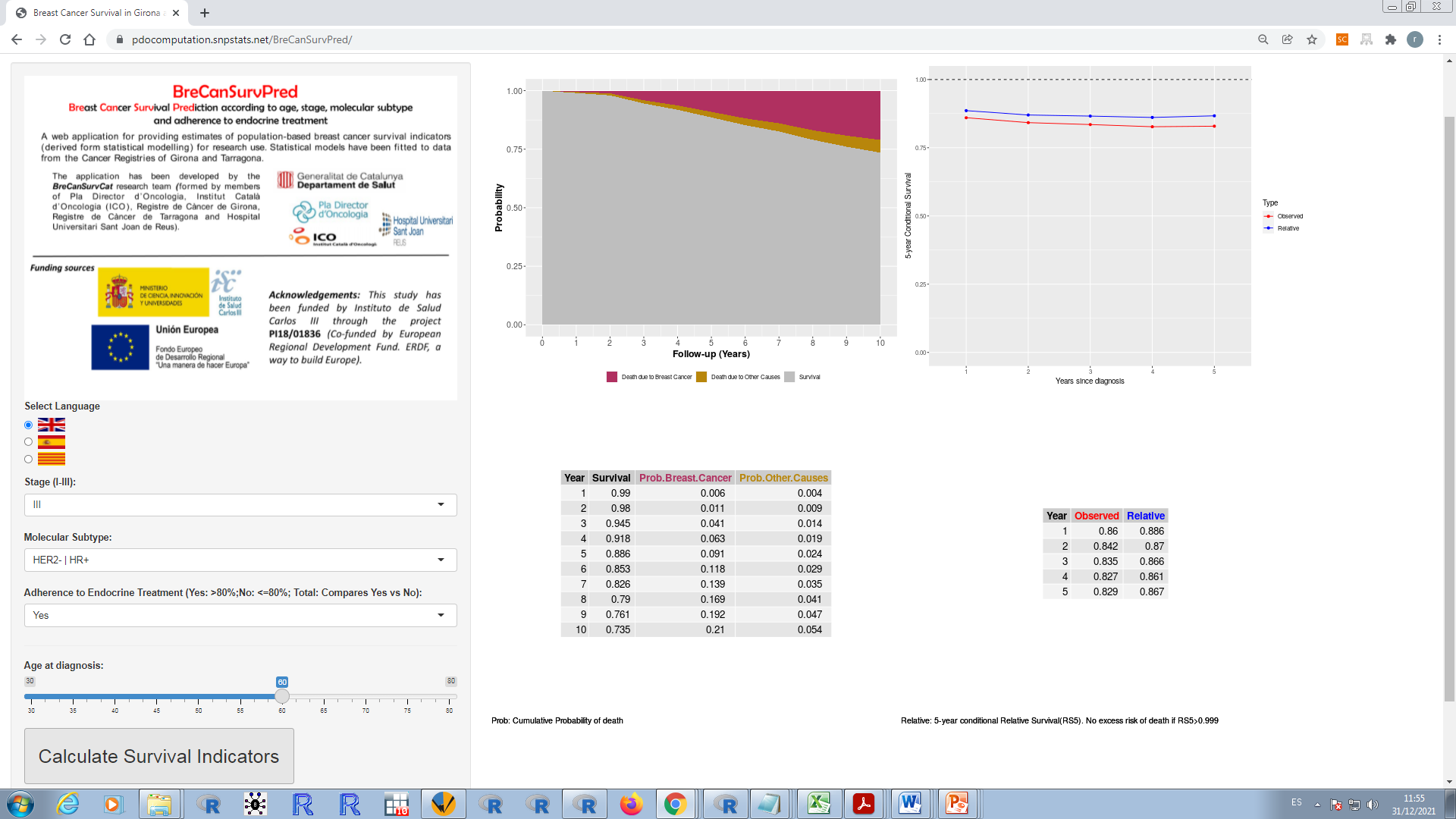
**Figure S4.** Snapshot of the web-based survival prediction application BreCanSurvPred. This snapshot demonstrates the probabilities of survival and death as well as the 5-year conditional probabilities of observed survival and relative survival for a 60-year-old patient who was *adherent* to endocrine therapy and diagnosed with molecular subtype HER2- / HR + in stage III. These probabilities are calculated up to 10 years after BC diagnosis.
